# Study of correlation between the NAT2 phenotype and genotype status among Greenlandic Inuit

**DOI:** 10.17179/excli2018-1671

**Published:** 2018-11-02

**Authors:** Emilie Birch Kristensen, Victor Yakimov, Karen Bjorn-Mortensen, Bolette Soborg, Anders Koch, Mikael Andersson, Kasper Birch Kristensen, Sascha Wilk Michelsen, Line Skotte, Anne Ahrendt Bjerregaard, Meinolf Blaszkewicz, Klaus Golka, Jan G. Hengstler, Bjarke Feenstra, Mads Melbye, Frank Geller

**Affiliations:** 1 Department of Epidemiology Research, Statens Serum Institut, Copenhagen, Denmark; 2 Ilisimatusarfik, University of Greenland, Nuuk, Greenland; 3 Leibniz Research Centre for Working Environment and Human Factors (IfADo), TU Dortmund, Germany; 4 Department of Clinical Medicine, University of Copenhagen, Denmark; 5 Department of Medicine, Stanford University School of Medicine, 300 Pasteur Drive, Stanford, CA, USA

**Keywords:** N-acetyltransferase 2, Greenland, NAT2 genotype status, NAT2 enzyme activity, caffeine test, isoniazid

## Abstract

*N*-acetyltransferase 2 (NAT2) is the main enzyme metabolizing isoniazid and genotype-based treatment has been studied for years without becoming common practice. To investigate whether genotype-based isoniazid treatment is feasible in Greenland, we sequenced the coding sequence of *NAT2 *and determined the NAT2 enzyme-activity by caffeine test.

No additional genetic variants were identified in the coding sequence of *NAT2*, so that genotype status in 260 study participants could be assessed by a well-established 7-SNP panel. Studying the enzyme activity by the ratio of the two caffeine metabolites AFMU and 1X in 260 participants showed a high rate of slow phenotypes with intermediate or rapid genotype. These misclassifications were mainly observed in urine samples with pH<3, a deviation from the standard protocol due to the field work character of the study, where immediate pH adjustment to pH=3.5 was not possible. We excluded these samples. For the remaining 143 individuals with pH>3, we observed a moderate level of discrepancies (19 of the 116 individuals with intermediate or rapid genotype status having a slow phenotype). Further investigation showed that drinking coffee and not tea or cola was the most important factor for high levels of both metabolites.

The concordance between phenotype and genotype status with regard to slow metabolism supported the recommendation of lower isoniazid doses in individuals with slow genotype status in order to avoid liver injury, a frequent side effect. The phenotypical variation observed for individuals with intermediate or rapid genotype status warrants further research before increased dosing of isoniazid can be recommended.

## Abbreviations

NAT2: *N*-acetyltransferase 2; TB: tuberculosis; SNP: single nucleotide polymorphism; 7-SNP panel: panel of seven SNPs in the coding sequence of *NAT2* often used to determine the genotype status; IfADo: Leibniz Research Centre for Working Environment and Human Factors; AFMU: 5-acetylamino-6-formylamino-3-methyluracil; 1X: 1-methylxanthine; IQR: inter quartile range; DQS: Dietary Quality Score; OR: odds ratio; 95 %CI: 95 % confidence interval

## Introduction

*N*-acetyltransferase 2 (NAT2) is a well-studied acetylating enzyme involved in the metabolism of drugs used in the treatment of e.g. tuberculosis (TB), AIDS-associated diseases and hypertension (McDonagh et al., 2014[22]; Weber, 1985[37]). NAT2 acetylates the drugs in the liver prior to excretion at a genetically determined rate. Variation in NAT2 activity has also been linked to the risk of cancer due to its role in the metabolism of xenobiotics (Figueroa et al., 2014[12]; Hein, 2002[15]; Marcus et al., 2000[21]; Rothman et al., 2010[26]; Tian et al., 2014[34]). 

The *NAT2* gene is located on chromosome 8p22 and has an 873 base pair coding sequence (Blum et al., 1990[5]). The coding exon of *NAT2* is polymorphic and frequencies of allelic variants vary between populations of different geographic and ethnic origins (Sabbagh et al., 2011[28]). Currently, 45 variants in the coding sequence have been described. Most common variants are functionally relevant and associated with reduced enzyme activity, whereas the haplotype containing the reference alleles is associated with rapid enzyme activity. Thus, haplotypes derived from *NAT2* genotypes are either associated with slow or rapid enzyme activity, resulting in three different outcomes for *NAT2* genotype status: slow (two slow haplotypes), intermediate (one slow and one rapid haplotype) and rapid (two rapid haplotypes) (Hein and Doll, 2012[16]; Marchand et al., 1996[20]). A panel of seven single nucleotide polymorphisms (SNPs) in the coding sequence is often used to determine the genotype status (7-SNP panel) (Hein and Doll, 2012[16]) whereas analysis of urinary metabolites after caffeine administration (caffeine test) is considered the gold standard for measuring the enzyme activity and assessing NAT2 phenotype status (Bolt et al., 2005[6]; Grant et al., 1984[14]). A high correlation between the detected phenotype status and the underlying genotype status has been demonstrated in several studies (Bolt et al., 2005[6]; Butcher, 2002[8]; Cascorbi et al., 1995[9]; Parkin et al., 1997[24]; Tang et al., 1991[33]; Zhao et al., 2000[39]). 

Around 4000 years ago, the first humans came from the North American Arctic to Greenland and populated the island in several migration waves (Raghavan et al., 2014[25]). Later immigration from Scandinavia over the last 300 years resulted in an additional European genetic component in the Greenlandic population. Recent studies have shown characteristic variants in metabolic genes in Greenlandic Inuit; a *TBC1D4* variant has been associated with type 2 diabetes and a *CPT1A* mutation associated with fatty acid metabolism (Moltke et al., 2014[23]; Skotte et al., 2017[30]). It is therefore likely that Inuit hold other specific gene variants. We recently used the 7-SNP panel to determine the *NAT2* genotype status among 1,556 Greenlandic individuals (Geller et al., 2016[13]). Our analyses showed a high frequency (36.4 %) of the rapid genotype status with a positive correlation between rapid genotype status and degree of Inuit ancestry.

Greenland has a high incidence of TB and isoniazid is used for both treatment and prevention of TB. A potential serious side effect of isoniazid treatment is liver injury (Azuma et al., 2012[1]; Kinzig-Schippers et al., 2005[19]; Wang et al., 2012[36]), affecting mainly individuals with slow phenotype status (Du et al., 2013[11]). Recent studies on the pharmacokinetics of isoniazid suggested that individualizing treatment dose according to *NAT2* genotype status could improve both the safety and efficacy of treatment (Azuma et al., 2012[1]; Jung et al., 2015[18]). If a high proportion of the Inuit population presents with rapid enzyme activity, the response to TB treatment and prevention might be lower than in other populations. 

The objective of the current study was to investigate whether a genotype-based treatment with different doses of isoniazid can be recommended for Greenland. Therefore, we sequenced the coding region of *NAT2* in 260 participants to search for additional known or Inuit-specific sequence variants associated with NAT2 enzyme activity. Afterwards, we compared the *NAT2* genotype status with phenotype status determined by the caffeine test. 

## Material and Methods

### Subjects

Greenland is a self-governed part of Denmark. In 2013, we recruited individuals from seven towns in North, South, East and West Greenland for a genetic study and investigated *NAT2* genotypes for the 7-SNP panel in 1,556 Greenlandic individuals (Geller et al., 2016[13]). For the current study, we re-invited all study participants from Sisimiut (n=374), Tasiilaq (n=167) and Nanortalik (n=168). Two weeks prior to enrolment, potential participants received a letter of invitation and, if possible, were contacted by phone. Participants were asked to empty their bladders and consume a caffeinated beverage at home. Then participants came to the local hospital, gave written informed consent and were enrolled. A venous blood sample was drawn and later a urine sample was collected. While waiting, study participants completed a standardized self-administered questionnaire covering information on smoking, alcohol habits, diet, health and lifestyle (for details see Supplementary Information). Basic variables including medication, emptied bladder, beverage (coffee or tea/cola), number of cups drunk, time to urination, and food consumed during waiting time were asked from each participant. TB is a mandatory notifiable disease in Greenland and all cases are reported to the tuberculosis registry, maintained by the National Board of Health. Information on TB diagnosis was obtained through the tuberculosis registry. Treatment history for TB cases was evaluated through medical records. Additional information was obtained from questionnaires filled out by 88 % of the participants during the time between giving blood and urination.

### Sequencing and determination of NAT2 haplotypes

After blood sampling into a 9 ml EDTA tube, plasma and buffy coat were separated and stored at -20 °C before transportation to Statens Serum Institut for DNA extraction. DNA was isolated from leukocytes in the buffy coat using Chemagic STAR DNA Buffy Coat kit BC200 (HAMILTON®). Double strand sequencing of the coding region of exon 2 of *NAT2* (873 bp) using Sanger technology took place at Eurofins MWG Operon, Ebersberg, Germany.

Sequence data for all 260 participants were analyzed using NovoSNP3.0.1 (Weckx et al., 2005[38]) and compared with the genotypes from the previous study (Geller et al., 2016[13]). Two discrepancies (0.13 %) between the genotype calls and the sequence calls were resolved by visual inspection of the sequence data and selecting the genotypes from sequencing. Haplotypes were estimated from the genotypes of the seven tagging SNPs with the algorithm implemented in the software PHASE (version 2.1.1) (Stephens and Donnelly, 2003[31]).

### Caffeine test

NAT2 enzyme activity was measured using a caffeine test suggested by Grant et al. (1984[14]) and validated by the German Research Foundation (Blaszkewicz, 2002[3]). After emptying their bladder, the participants consumed 1-3 cups of either coffee, tea or cola. Approximately 1-3 hours after intake, a urine sample was collected from each participant. There was no need for fasting prior to urine collection. To adjust the urine simultaneously to an acidic pH in the pH range 3-5, samples were immediately filled into 10 mL sealable plastic tubes prepared with 38 mg dissolved sodium hydrogen sulfate monohydrate, a procedure experimentally established with 20 urine samples at the Leibniz Research Centre for Working Environment and Human Factors (IfADo). Due to the relative instability of one caffeine metabolite (Tang et al., 1983[32]), samples were subsequently stored at -20 °C and transported in ice-boxes to the IfADo for analysis. Here, pH measurements were performed with a calibrated pH meter with a glass membrane. Creatinine in urine was measured with photometric microplate reader according to the Jaffe method (Blaszkewicz and Liesenhoff‐Henze, 2012[4]). The secondary caffeine metabolites 5-acetylamino-6-formylamino-3-methyluracil (AFMU) and 1-methylxanthine (1X) in urine were determined using high performance liquid chromatography separation with UV detection. The AFMU/1X molar ratio was used as determinant of phenotype status. All measurements were done in duplicates and the mean of the two ratios was used as an indicator of phenotype. A cut-off AFMU/1X of 0.85 distinguished between slow and intermediate/rapid phenotype status (Bolt et al., 2005[6]; Braz Vieira da Silva Pontes et al., 1993[7]). 

### Statistical analyses

Median and inter quartile range (IQR) of AFMU/1X were calculated for the basic variables ascertained at sample collection and the health and lifestyle components from the questionnaire. We analyzed the natural logarithm of the metabolite measurements for AFMU, 1X and their ratio by multiple regression models in R. Factors were stepwise added to the model based on an inclusion p-value of 0.05. Similarly, we analyzed the binary variable misclassification by logistic regression in the subgroup with intermediate or rapid genotype status.

## Results

Of 709 potential participants, 265 (37 %) individuals accepted to participate, and of these 260 (70 % female) had the coding region of *NAT2* sequenced and their AFMU and 1X levels determined. Genotype status was assessed as rapid, intermediate or slow, depending on the presence of 0, 1 or 2 slow haplotypes, respectively.

### Sequencing and determination of NAT2 haplotypes and genotype status

Apart from the six SNPs of the 7-SNP-panel that were polymorphic in Greenland in our previous study (Geller et al., 2016[13]), no variation in the coding sequence of *NAT2* was detected. The frequencies of the different *NAT2* haplotypes are given in Table 1(Tab. 1). The rapid haplotype *4 was most frequent in our study group (64 %), and the slow haplotypes were largely represented by *5B and *6A with 19 % and 14 %, respectively. For additional analyses, we also investigated the subgroup of ultra-slow haplotypes (*6A or *7B) (Ruiz et al., 2012[27]; Selinski et al., 2013[29]), for which an increased risk of hepatotoxicity in TB patients has been reported (Higuchi et al., 2007[17]). Ultra-slow haplotypes were observed at a frequency of 15 % in our study group. The genotype distribution of the six SNPs is given in Supplementary Table 1. 

### Quality control of caffeine test

AFMU/1X measured by the caffeine test ranged from 0.004 to 6.34 (median 0.94, IQR: 0.28-1.35) and 121 participants (47 %) were classified with slow phenotype status. Genotypically, only 16 % had a status associated with slow metabolism. To further investigate these misclassifications, we looked for factors influencing the metabolite levels and the ratio of them. It is known that an adjustment of urine to acidic pH is crucial for accurate measurements and it is recommended to adjust pH to 3.5 manually. However, in this field study samples were immediately filled into 10 mL plastic tubes containing 38 mg dissolved sodium hydrogen sulfate monohydrate. Measurements of pH in the laboratory varied from 1.75 to 6.56. Statistical analysis revealed an association between pH values and log (AFMU/1X), r=0.32, p=1.0×10^-7^, which could explain AFMU/1X values resulting in misclassifications. To investigate whether this association was limited to a certain range of pH values, we split the samples in pH<3, pH between 3 and 4, and pH>4. Supplementary Table 2 shows the 2×3 tables for the three categories, with the 117 individuals in the group with pH<3 having 51 % misclassification vs. 13 % in the remaining samples. We observed no increased rate of misclassification in the group with pH>4. As we had noticed discrepancies between phenotype and genotype status early on in the study, we had asked some participants to provide a second urine sample some days (median 6, range 2-43 days) after the first sample and 15 participants showed up a second time (Supplementary Table 3) all other analyses are based on data related to participants' first urine sample. Nine of ten misclassifications occur in urine samples with pH<3, including all four samples from two participants with slow AFMU/1X in both measurements despite intermediate/rapid genotype status. Given this evidence that urine samples with pH<3 do not provide reliable metabolite measurements for NAT2 phenotyping, we excluded all 117 participants with pH<3 in their first samples from further analyses. The pH values in women (mean 3.19, 95 %CI: 3.05-3.33) were significantly lower than in men (mean 3.68, 95 %CI: 3.46-3.91), thus females accounted for 81 % of the excluded participants compared to 70 % of the initial study group. In the remaining 143 participants pH values and log (AFMU/1X) were no longer correlated, r=0.04, p=0.60.

### Distribution of AFMU/1X

**Figure 1:**
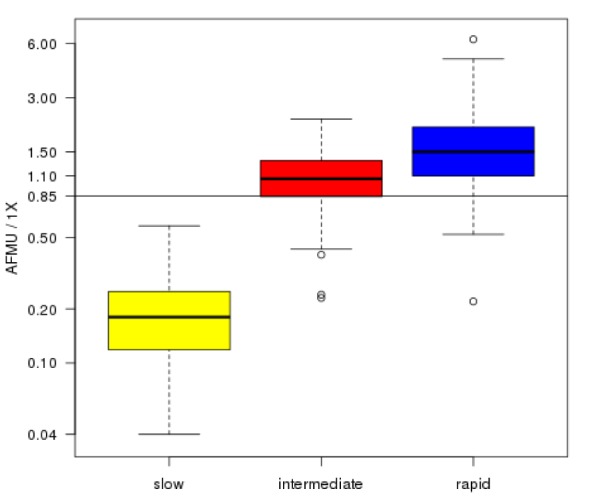
Distribution of AFMU/1X according to genotype status for 143 study participants

Figure 1(Fig. 1) illustrates the distribution of the actual AFMU/1X over the three genotype status groups in the remaining 143 participants, showing the wide range of ratios observed for intermediate and rapid genotype status. Supplementary Table 2 presents AFMU/1X median and IQR for all basic and extended variables under investigation. The distribution of phenotype and genotype status is shown in Table 2(Tab. 2). All 27 individuals with a slow genotype status had a slow phenotype status (100 % concordance), whilst 84 % (97 of 116) of individuals with an intermediate or rapid genotype status presented with the expected intermediate/rapid phenotype.

### Analysis of metabolites AFMU, 1X and AFMU/1X

To identify factors associated with the 19 observed discrepancies between phenotype and genotype status, we built multiple regression models for the two metabolites 1X, AFMU, and AFMU/1X. The three outcomes had skewed distributions; so we applied a natural log-transformation to the values before analysis. 

We investigated models with genotype status, laboratory measurements (pH, creatinine), basic information obtained at urine sample collection and information from questionnaires as explanatory variables. The final models from the stepwise regression for 1X, AFMU and AFMU/1X are given in Table 3(Tab. 3). For AFMU and 1X the same six variables met the significance threshold of P≤0.05. The variable indicating whether individuals drank coffee and not tea or cola was associated with a large increase in 1X and AFMU and explained about 32 % of the variance in both measurements. The difference in the effect estimates was small, and so coffee did not have a significant effect on AFMU/1X. The effect of genotype status was stronger for AFMU (15 % variance explained) than for 1X (5 % variance explained). Creatinine levels had similar effects for both metabolites, explaining up to 10 % of the variance. The remaining factors - smoking, age and diet - explained between 2 % and 7 % of the variation in the metabolite levels. Increased metabolite values were observed for smokers, older individuals and individuals with a higher (healthier) Dietary Quality Score (DQS) (Toft et al., 2006[35]). Given our data, the effect of age was not a consequence of oral contraceptive use: ten women had answered that they were on oral contraceptives when asked for their medication and models including oral contraceptives had very similar effect estimates for age (all P≤0.05 for age), whereas the low powered analysis of oral contraceptive use did not result in any P≤0.05. For AFMU/1X, including the number of ultra-slow haplotypes *6A and *7B further improved the fit of the model. In total, the genotypes explain 72 % of the variance. The only other factor with a significant effect was age. Supplementary Table 5 provides a model for AFMU/1X also including the other four variables from the models for the individual metabolites, confirming that they have no significant effect on the ratio (all P>0.2). 

### Analysis of misclassification

The 13 % of misclassification observed is still somewhat higher than rates observed in similar studies. Therefore, we investigated misclassification as a binary outcome by logistic regression. All misclassifications occurred in individuals presenting with slow phenotype status and intermediate/rapid genotype status. No misclassification occurred in the group with slow genotype status and these individuals were not included in the model. Intermediate genotype status was associated with lower AFMU/1X and was therefore included in the model as risk factor for misclassification (OR=5.87, 95 %CI: 1.71-20.09, P=0.0049). Emptying the bladder before beverage consumption was the only other factor reducing the odds ratio of misclassification significantly (OR=5.17, 95 %CI: 1.60-16.74, P=0.0061).

The metabolite 1X was negatively associated with misclassification (r=-0.23, P=0.011, based on log-values), indicating that lower values of 1X more often lead to misclassifications, even though 1X factors in as denominator. As some measurements of the reference metabolite 1X were rather low, values of 1X might also be an indicator of how well the caffeine test in a participant went. In participants with 1X above the median of 98.25, there was no association with misclassification (r=0.01, P=0.96). 

### TB history related to NAT2 phenotype and genotype status 

Sixteen of the 260 participants had a history of active TB before participating in the study, all received standard treatment, including 300 mg isoniazid per day over six months according to WHO recommendations. All but one case came from Tasiilaq, East Greenland, a region with a particularly high TB incidence (Bjorn-Mortensen et al., 2015[2]). Genotype status was distributed as two slow, four intermediate and ten rapid. Reviewing their medical records revealed no cases of liver injury or treatment failure, i.e. all 13 cases with a positive culture test (three not tested) proved to be sensitive to standard treatment.

## Discussion

This study aimed to determine NAT2 phenotype status and to compare it to *NAT2* genotype status in 260 study participants from three towns in Greenland. Sequencing of the coding region of *NAT2* detected no additional variation, so that the previously analyzed six polymorphic SNPs of the 7-SNP-panel were sufficient to assess *NAT2* genotype status. NAT2 phenotype status was studied by the caffeine test and determined by the ratio of the metabolites AFMU and 1X. However, the concordance between genotype and phenotype status was rather poor and splitting the cohort in three groups showed that it was mainly the group with pH<3 where AFMU/1X was low for individuals with intermediate/rapid genotype status. In the context of this field study, the recommended manual adjustment to pH 3.5 was not feasible and we worked with a standard concentration of sodium hydrogen sulfate monohydrate. Here, the metabolite measurements appear relatively stable in the pH range from 3 to 6, so that a strategy filling the sampled urine of a participant in two plastic tubes with two different concentrations of sodium hydrogen sulfate monohydrate might guarantee a urine sample with pH between 3 and 5 for each participant in future studies.

Analyses based on the urine metabolites were restricted to samples with pH>3, leaving 143 participants. Here, 13 % showed a slow phenotype status despite intermediate/rapid genotype status. The observed correlation between NAT2 phenotype and genotype status is slightly lower than what has been reported in most other studies among different ethnic groups (Bolt et al., 2005[6]; Butcher, 2002[8]; Cascorbi et al., 1995[9]; Parkin et al., 1997[24]; Tang et al., 1991[33]; Zhao et al., 2000[39]). However, substantial discrepancies have also been reported. A study comparing the NAT2 phenotype and genotype status between Swedes and Koreans showed a significantly different enzyme activity between the two groups, even when comparing the same *NAT2* haplotypes (Djordjevic et al., 2012[10]). Another study reported both slow and rapid phenotypes among individuals with intermediate genotype status (Bolt et al., 2005[6]). 

Investigating the two metabolites 1X and AFMU showed increased urine levels when caffeine was administered via coffee instead of cola or tea. The choice of coffee explained about 32 % of the variance of both metabolites. Future studies could consider administering 100 mg caffeine tablets to ensure a sufficient caffeine intake. The genotype had a stronger effect on AFMU than on 1X. Increased levels of the metabolites were also observed for participants who smoked, were older, had higher creatinine levels, or were on a healthier diet according to the DQS. The unexpected association between age and the metabolite levels might not be caused directly by age, it is well possible that in the Greenlandic study group some relevant factors were correlated with age but not measured. Further studies are needed to follow-up on this. Ultra-slow haplotypes (*6A and *7B) had a frequency of 15 % in this study, and the number of ultra-slow haplotypes significantly reduced AFMU/1X in regression models already accounting for slow and intermediate genotype status. In our study, *NAT2* genotypes explain 72 % in the variability of AFMU/1X, which is not too far from the reported 88 % explained in isoniazid clearance (Kinzig-Schippers et al., 2005[19]).

Misclassification was significantly more frequent in cases where the bladder was not emptied before drinking the beverage (OR=5.17, 95 %CI: 1.60-16.74). Together with the observation that the levels of the individual metabolites depend on several factors, it seems reasonable that deviations from a perfect trial set-up explain the remaining misclassifications. 

In conclusion, our study showed that in Greenland NAT2 genotype status can be determined via six SNPs from the 7-SNP-panel. The concordance between the NAT2 phenotype and genotype status was corrupted in urine samples with a pH<3 and levels of the metabolites AFMU and 1X were lower when participants drank cola or tea instead of coffee. Therefore, future field studies should ensure sufficient caffeine intake and carefully monitor the pH values of the urine samples. The considerable variation in AFMU/1X observed in individuals with intermediate and rapid genotype status precludes increased isoniazid dose-regimes for these individuals before differences in enzyme activity are better understood. In contrast, all individuals with slow genotype status had slow enzyme activity and may very well benefit from a reduced isoniazid dose regime resulting in fewer adverse events and possibly better compliance. Thus, our findings justify decreased isoniazid doses for TB patients with slow genotype status when at the same time treatment effectiveness is carefully monitored. Future studies on plasma levels of acetyl-isoniazid in Greenlandic individuals with rapid genotype status are needed to justify increased doses of isoniazid for them.

## Ethical considerations

The study fulfilled the Helsinki Declaration II. The commission for Scientific Research in Greenland (approval No. 2015-05) and the Danish Data Protection Agency approved the study. 

## Acknowledgements

The authors thank all volunteers for their participation in this study and the staff at the district hospitals in Sisimiut, Tasiilaq and Nanortalik for welcoming us. Additional thanks to the translators Ane Møller (Sisimiut), Abia Kuitse (Tasiilaq), Helga Dorph Nielsen and Judithe Berthelsen (Nanortalik) for their help in conducting the fieldwork. 

The study was supported by grants from the Danish Council for Independent Research (DFF), Sundhedspuljen (Grønlands Sundhedsvidenskabelige Forskningsråd), Torben og Alice Frimodts Fond, Augustinusfondet, Fonden til Lægevidenskabens Fremme (The Maersk Foundation), NunaFonden, The Højmosegård grant and Frimodt-Heinike Fonden. Bjarke Feenstra was supported by an Oak Foundation fellowship. This research has been conducted using the Danish National Biobank resource. The Danish National Biobank is supported by the Novo Nordisk Foundation. The sponsors played no role in the study design, in conducting the study or in writing the article. 

## Conflict of interest

The authors declare no conflicts of interest.

## Supplementary Material

Supplementary information

## Figures and Tables

**Table 1:**
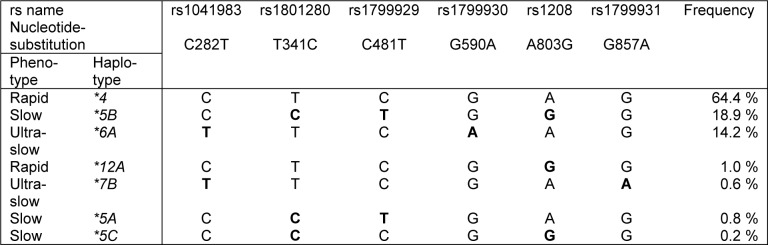
Frequencies of *NAT2* haplotypes in 260 study participants. Results based on the six SNPs from the 7-SNP panel that were polymorphic in the study group.

**Table 2:**
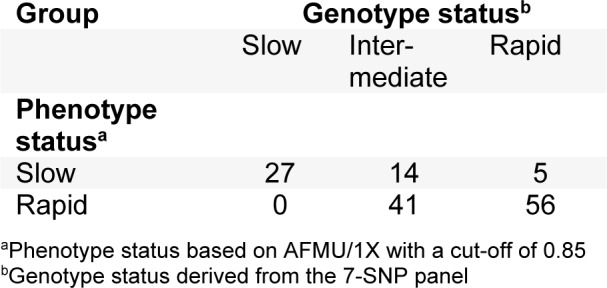
Distribution of phenotype and genotype status for 143 study participants

**Table 3:**
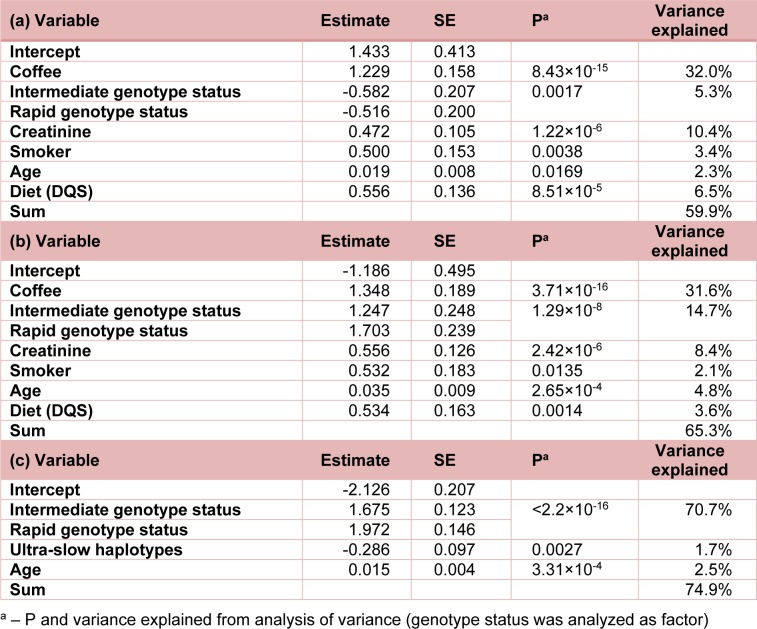
Multiple linear regression model for (a) log(1X), N=111 due to missing values for some variables, (b) log(AFMU), N=111 due to missing values for some variables, (c) log(AFMU/1X), N=143.

## References

[1] Azuma J, Ohno M, Kubota R, Yokota S, Nagai T, Tsuyuguchi K (2012). NAT2 genotype guided regimen reduces isoniazid-induced liver injury and early treatment failure in the 6-month four-drug standard treatment of tuberculosis: A randomized controlled trial for pharmacogenetics-based therapy. Eur J Clin Pharmacol.

[2] Bjorn-Mortensen K, Andersen AB, Koch A, Ladefoged K, Lillebaek T, Michelsen SW (2015). Tuberculosis outbreak in East Greenland: groups at risk in an isolated arctic setting. Eur Respir J.

[3] Blaszkewicz M (2002). N-Acetyltransferase 2 (phenotyping: caffeine test) [Biomonitoring Methods, 2004]. MAK-Collect Occup Health Saf [Internet]. http://onlinelibrary.wiley.com/doi/10.1002/3527600418.bi0nat2phne0009/abstract.

[4] Blaszkewicz M, Liesenhoff-Henze K (2012). Creatinine in urine [Biomonitoring Methods, 2010]. MAK-Collection Occup Health Saf [Internet]. https://onlinelibrary.wiley.com/doi/10.1002/3527600418.bi6027urie0012.

[5] Blum M, Grant DM, McBride W, Heim M, Meyer UA (1990). Human arylamine N-acetyltransferase genes: isola-tion, chromosomal localization, and functional ex-pression. DNA Cell Biol.

[6] Bolt HM, Selinski S, Dannappel D, Blaszkewicz M, Golka K (2005). Re-investigation of the concordance of hu-man NAT2 phenotypes and genotypes. Arch Toxicol.

[7] Braz Vieira da Silva Pontes Z, Vincent-Viry M, Gueguen R, Galteau MM, Siest G (1993). Acetylation pheno-types and biological variation in a French Caucasian population. Clin Chem Lab Med.

[8] Butcher NJ (2002). Pharmacogenetics of the arylamine N-acetyltransferases. Pharmacogenom J.

[9] Cascorbi I, Drakoulis N, Brockmöller J, Maurer A, Sperling K, Roots I (1995). Arylamine N-acetyltransferase (NAT2) mutations and their allelic linkage in unrelat-ed Caucasian individuals: correlation with phenotypic activity. Am J Hum Genet.

[10] Djordjevic N, Carrillo JA, Roh H-K, Karlsson S, Ueda N, Bertilsson L (2012). Comparison of N-acetyltransferase-2 enzyme genotype-phenotype and xanthine oxidase enzyme activity between Swedes and Koreans. J Clin Pharmacol.

[11] Du H, Chen X, Fang Y, Yan O, Xu H, Li L (2013). Slow N-acetyltransferase 2 genotype contributes to anti-tuberculosis drug-induced hepatotoxicity: a meta-analysis. Mol Biol Rep.

[12] Figueroa JD, Ye Y, Siddiq A, Garcia-Closas M, Chat-terjee N, Prokunina-Olsson L (2014). Genome-wide as-sociation study identifies multiple loci associated with bladder cancer risk. Hum Mol Genet.

[13] Geller F, Soborg B, Koch A, Michelsen SW, Bjorn-Mortensen K, Carstensen L (2016). Determination of NAT2 acetylation status in the Greenlandic popula-tion. Arch Toxicol.

[14] Grant D, Tang B, Kalow W (1984). A simple test for acetyla-tor phenotype using caffeine. Br J Clin Pharmacol.

[15] Hein DW (2002). Molecular genetics and function of NAT1 and NAT2: role in aromatic amine metabolism and carcinogenesis. Mutat Res Mol Mech Mutagen.

[16] Hein DW, Doll MA (2012). Accuracy of various human NAT2 SNP genotyping panels to infer rapid, intermediate and slow acetylator phenotypes. Pharmacogenomics.

[17] Higuchi N, Tahara N, Yanagihara K, Fukushima K, Suyama N, Inoue Y (2007). NAT2*6A, a haplotype of the N-acetyltransferase 2 gene, is an important bi-omarker for risk of anti-tuberculosis drug-induced hepatotoxicity in Japanese patients with tuberculosis. World J Gastroenterol.

[18] Jung JA, Kim T-E, Lee H, Jeong B-H, Park HY, Jeon K (2015). A proposal for an individualized pharmaco-genetic-guided isoniazid dosage regimen for patients with tuberculosis. Drug Des Devel Ther.

[19] Kinzig-Schippers M, Tomalik-Scharte D, Jetter A, Scheidel B, Jakob V, Rodamer M (2005). Should we use N-acetyltransferase type 2 genotyping to personalize isoniazid doses?. Antimicrob Agents Chemother.

[20] Marchand LL, Sivaraman L, Franke AA, Custer LJ, Wilkens LR, Lau AF (1996). Predictors of N-acetyltransferase activity: should caffeine phenotyping and NAT2 genotyping be used interchangeably in epidemiological studies?. Cancer Epidemiol Biomarkers Prev.

[21] Marcus PM, Vineis P, Rothman N (2000). NAT2 slow acety-lation and bladder cancer risk: a meta-analysis of 22 case-control studies conducted in the general popula-tion. Pharmacogenetics.

[22] McDonagh EM, Boukouvala S, Aklillu E, Hein DW, Altman RB, Klein TE (2014). PharmGKB summary: very important pharmacogene information for N-acetyltransferase 2. Pharmacogenet Genom.

[23] Moltke I, Grarup N, Jørgensen ME, Bjerregaard P, Treebak JT, Fumagalli M (2014). A common Green-landic TBC1D4 variant confers muscle insulin re-sistance and type 2 diabetes. Nature.

[24] Parkin DP, Vandenplas S, Botha FJ, Vandenplas ML, Seifart HI, van Helden PD (1997). Trimodality of isoni-azid elimination: phenotype and genotype in patients with tuberculosis. Am J Respir Crit Care Med.

[25] Raghavan M, DeGiorgio M, Albrechtsen A, Moltke I, Skoglund P, Korneliussen TS (2014). The genetic pre-history of the New World Arctic. Science.

[26] Rothman N, Garcia-Closas M, Chatterjee N, Malats N, Wu X, Figueroa JD (2010). A multi-stage genome-wide association study of bladder cancer identifies multiple susceptibility loci. Nat Genet.

[27] Ruiz JD, Martínez C, Anderson K, Gross M, Lang NP, García-Martín E (2012). The differential effect of NAT2 variant alleles permits refinement in phenotype inference and identifies a very slow acetylation genotype. PLoS ONE.

[28] Sabbagh A, Darlu P, Crouau-Roy B, Poloni ES (2011). Ar-ylamine N-Acetyltransferase 2 (NAT2) genetic diver-sity and traditional subsistence: a worldwide popula-tion survey. PLoS ONE.

[29] Selinski S, Blaszkewicz M, Ickstadt K, Hengstler JG, Golka K (2013). Refinement of the prediction of N-acetyltransferase 2 (NAT2) phenotypes with respect to enzyme activity and urinary bladder cancer risk. Arch Toxicol.

[30] Skotte L, Koch A, Yakimov V, Zhou S, Søborg B, Andersson M (2017). CPT1A missense mutation asso-ciated with fatty acid metabolism and reduced height in Greenlander. Circ Cardiovasc Genet.

[31] Stephens M, Donnelly P (2003). A Comparison of Bayesian methods for haplotype reconstruction from population genotype data. Am J Hum Genet.

[32] Tang BK, Grant DM, Kalow W (1983). Isolation and identifi-cation of 5-acetylamino-6-formylamino-3-methyluracil as a major metabolite of caffeine in man. Drug Metab Dispos.

[33] Tang B-K, Kadar D, Qian L, Iriah J, Yip J, Kalow W (1991). Caffeine as a metabolic probe: Validation of its use for acetylator phenotyping. Clin Pharmacol Ther.

[34] Tian F-S, Shen L, Ren Y-W, Zhang Y, Yin Z-H, Zhou B-S (2014). N-acetyltransferase 2 gene polymorphisms are associated with susceptibility to cancer: a meta-analysis. Asian Pac J Cancer Prev. APJCP.

[35] Toft U, Kristoffersen LH, Lau C, Borch-Johnsen K, Jørgensen T (2006). The Dietary Quality Score: validation and association with cardiovascular risk factors: the Inter99 study. Eur J Clin Nutr.

[36] Wang P-Y, Xie S-Y, Hao Q, Zhang C, Jiang B-F (2012). NAT2 polymorphisms and susceptibility to anti-tuberculosis drug-induced liver injury: a meta-analysis. Int J Tuberc Lung Dis.

[37] Weber WW (1985). N-acetylation pharmacogenetics. Pharmacol Rev.

[38] Weckx S, Del-Favero J, Rademakers R, Claes L, Cruts M, De Jonghe P (2005). NovoSNP, a novel computat-ional tool for sequence variation discovery. Genome Res.

[39] Zhao B, Seow A, Lee EJD, Lee H-P (2000). Correlation be-tween acetylation phenotype and genotype in Chinese women. Eur J Clin Pharmacol.

